# Corneal Higher Order Aberrations in Granular, Lattice and Macular Corneal Dystrophies

**DOI:** 10.1371/journal.pone.0161075

**Published:** 2016-08-18

**Authors:** Yukari Yagi-Yaguchi, Takefumi Yamaguchi, Yumi Okuyama, Yoshiyuki Satake, Kazuo Tsubota, Jun Shimazaki

**Affiliations:** 1 Department of Ophthalmology, Ichikawa General Hospital, Tokyo Dental College, Chiba, Japan; 2 Department of Ophthalmology, Keio University School of Medicine, Tokyo, Japan; National Eye Institute, UNITED STATES

## Abstract

**Purpose:**

To evaluate the corneal higher-order aberrations (HOAs) in granular, lattice and macular corneal dystrophies.

**Methods:**

This retrospective study includes consecutive patients who were diagnosed as granular corneal dystrophy type2 (GCD2; 121 eyes), lattice corneal dystrophies type 1, type 3A (LCDI; 20 eyes, LCDIIIA; 32 eyes) and macular corneal dystrophies (MCD; 13 eyes), and 18 healthy control eyes. Corneal HOAs were calculated using anterior segment optical coherence tomography, and the correlations between HOAs and visual acuity were analyzed.

**Results:**

HOAs of the total cornea within 4 mm diameter were significantly larger in GCD2 (0.17 ± 0.35 μm), in LCDI (0.33 ± 0.27), LCDIIIA (0.61 ± 1.56) and in MCD (0.23 ± 0.18), compared with healthy controls (0.09 ± 0.02μm, all P < 0.01). HOAs of the total cornea within 6 mm diameter were significantly larger in GCD2 (0.32 ± 0.48), in LCDI (0.60 ± 0.46), LCDIIIA (0.83 ± 2.29) and in MCD (0.44 ± 0.24), compared with healthy controls (0.19 ± 0.06, all P < 0.001). In GCD2, there was no significant correlation between logMAR and HOAs (r = 0.113, P = 0.227). In MCD, LCDI and LCDIIIA, logMAR was positively significantly correlated with HOAs (r = 0.620 and P = 0.028, r = 0.587 and P = 0.007, r = 0.614 and P < 0.001, respectively).

**Conclusions:**

Increased HOAs occur in eyes with corneal dystrophies, especially in eye with LCD and MCD. Larger amount corneal HOAs are associated with poorer visual acuity in patients with LCD and MCD.

## Introduction

Hereditary corneal dystrophies are characterized by the occurrence of bilateral progressive opacities of the corneal stroma, leading to visual impairment and photophobia, as well as concomitant symptoms of ocular irritation and recurrent corneal erosions. Genotypic analyses have provided a clear understanding of the gene defect and resulting consequences of the mutant protein. Mutations in the transforming growth factor beta-induced (*TGFBI*) gene are responsible for stromal dystrophies, such as lattice corneal dystrophy (LCD) and granular corneal dystrophy (GCD).[1] Mutations in corneal carbohydrate sulfotransferase 6 (*CHST6*) gene are responsible for macular corneal dystrophy (MCD).[2] The clinical manifestations vary significantly, depending on the anatomical location, and the pattern and severity of the protein deposition. LCD characterized by a network of multiple branching refractive lines or fusiform corneal opacities due to amyloid deposition in the anterior and deep-stromal levels.[1] GCD is characterized by multiple discrete granular white opacities (hyaline deposition) in the central anterior stroma.[3] MCD is characterized by diffuse stromal clouding due to the accumulation of glycosaminoglycans and central corneal thinning.[4]

The recent technological advances have been used to obtain images and precise information of the cornea and the anterior portion of the eyes.[5–7] In addition, a methodology for evaluating optical and visual functions has been developed and applied to normal eyes [8] and other ocular pathologies, such as dry eye disease, [9] corneal diseases, [10–12] cataract, [13] and vitreous surgeries.[14] Thus, wavefront analysis to quantify lower-order aberrations and higher-order aberrations (HOAs) has provided an explanation for the decrease of visual acuity and contrast sensitivity in normal and eyes after ocular surgeries. In these analyses, a larger HOAs is correlated with decreased visual function.[8, 11, 15]

Recently, we noticed the irregular posterior corneal surfaces in some patients with severe LCD, while the posterior surfaces in MCD and GCD were smooth with minimal irregularity. The irregular posterior corneal surfaces are supposed to result in an increase in total HOAs and degraded quality of vision.[16] However, to the best of our knowledge, the influence of HOAs on visual acuity is still poorly understood in eyes with corneal dystrophies. In the following study, we hypothesized that increased HOAs have adverse effects on the visual acuity in corneal dystrophies, although the reason for decreased visual acuity in these corneal dystrophies have been thought to be due to the stromal opacities. First, we calculated and characterized the corneal HOA profiles in GCD, LCD and MCD, and compared with HOAs in normal subjects. Second, we separately evaluate the correlation between visual acuity and HOAs in groups with GCD, LCD and MCD, with the aim of identifying the effect of HOAs on visual function in each group. Our results demonstrated that HOAs are increased in LCD, especially in lattice corneal dystrophy type 3A (LCDIIIA), compared with GCD and MCD. Furthermore, HOAs were inversely correlated with visual acuity in LCD and MCD, suggesting that HOAs degrade visual acuity together with stromal opacities in corneal dystrophies. These results show that the imaging of the corneal surface irregularities can provide clinicians with important information pertinent to diagnosis and decision making of surgical intervention, such as phototherapeutic keratectomy (PTK) and corneal transplantation.

## Patients and Methods

This retrospective consecutive study was performed in accordance with the Declaration of Helsinki and approved by the institutional Ethic Review Board of Tokyo Dental College Ichikawa General Hospital (I-15-51). Our Institutional Review Board waived the requirement for informed consent for this retrospective study. Patient data was anonymized before access and/or analysis.

### Patients

The database of Ichikawa General Hospital (MegaOak version 4.0, NEC Inc., Tokyo, Japan) contains detailed medical record data of 150,605 visits of 26,042 patients from January 2010 to July 2015. The dataset contains information regarding the age, gender, address, diagnosis, prescriptions and referral letters submitted by ophthalmologists. The database contains detailed records of all patients in Tokyo Dental College, Ichikawa General Hospital from 2002 onwards. Corneal dystrophies were registered as GCD, LCD or MCD in the database. We searched for evidence of corneal epithelial/stromal dystrophy or corneal stromal dystrophy based on the diagnosis using the keywords of “lattice corneal dystrophy”, “macular corneal dystrophy” and “granular corneal dystrophy” and found 93 patients with a diagnosis of “GCD”, 44 patients with a diagnosis of “LCD”, and 11 patients with a diagnosis of “MCD”. Two corneal specialists (YY and TY) checked all of the patients’ medical records and excluded patients with the following: a wrong diagnosis, epithelial defects, scarring, a history of corneal surgery such as PTK and corneal transplantation. One of the corneal specialists clinically diagnosed corneal dystrophies from patient histories, family histories and slit-lamp examinations, based on the international classification of corneal dystrophies (IC3D).[17] Genetic analyses were not conducted in the current study. In the end, 121 eyes of 63 patients with GCD type 2 (GCD2), 20 eyes of 11 patients with LCD type 1 (LCDI), 32 eyes of 20 patients with LCD type 3A (LCDIIIA), and 13 eyes of 7 patients with MCD were found and included in the current study. Eighteen eyes of 18 volunteer healthy subjects were included as the normal control (S1 Dataset).

Anterior segment optical coherence tomography (AS-OCT) (SS-1000, CASIA, Tomey, Nagoya, Japan) has been used since 2010 in Tokyo Dental College, Ichikawa General Hospital. AS-OCT the images were routinely taken in all patients with corneal dystrophies.

### Data Analysis

Routine examinations including slit-lamp microscopy, fundus examinations, best corrected visual acuity (BCVA), subjective spherical equivalent (SE) and subjective astigmatism were measured at the time of diagnosis for all patients. Visual acuity was measured using the standard Snellen chart, and BCVA with spectacle correction was recorded. The results were converted into logarithm of minimal angle resolution (logMAR) units. The extent of corneal opacity was graded by two masked observers (YO and TY) on the basis of the slit-lamp examination with a previously described system.[18] Grade 0 denoted clear or a trace haze, grade 1 denoted a mild opacity, grade 2 denoted a moderately dense opacity partially obscuring details of the iris, and grade 3 denoted severely dense opacity obscuring details of the intraocular structure.

### AS-OCT

The eyes of control subjects and patients with GCD, LCD and MCD were routinely examined using AS-OCT. All subjects were examined until at least two sets of excellent images were obtained. Sixteen rotating AS-OCT scans were used to reconstruct three-dimensional models of the entire corneal structure. The CASIA system (Tomey) corrected distortions in the AS-OCT images based on the refractive index of the anterior surface. A corneal specialist (YY) carefully checked all AS-OCT images to ensure that the surface digitalization recognized by the automated inbuilt software was correct. Zernike coefficients were calculated using Zernike analysis as previously reported.[6] In brief, the anterior and posterior corneal surfaces were reconstructed as a three-dimensional model from the corneal height data. The anterior, posterior and total corneal aberrations at the diameters of 4mm and 6mm were calculated separately with installed ray tracing software, version 5.1. The refractive indices of the cornea and aqueous humor were set to 1.376 and 1.336, respectively. The wavefront aberration was expanded with normalized Zernike polynomials up to the 8^th^ order. HOA was defined as the root mean square (RMS) of the 3^rd^- to 8^th^-order Zernike coefficients as follows.[19]

$$HOA=\sqrt{\sum_{j=3}^{20}{\left(Z_{j}\right)}^{2}}$$

$$Z_{j}=Z_{n}^{m}$$

$$j=\frac{n(n+2)+m}{2}$$

$$n=roundup\left[\frac{-3+\sqrt{9+8j}}{2}\right]$$

m=2j−n(n+2)

Spherical aberration (SA) was defined as the RMS of Z_4_° (spherical aberration) and Z_6_°(secondary spherical aberration). Coma aberration was defined as the RMS of Z_3_^-1^ and Z_3_^1^.

$${SA}^{}={\sqrt{Z_{4}^{0}{}^{{}^{2}}+Z_{6}^{0}{}^{{}^{2}}}}^{}$$

$${Coma}^{}={\sqrt{Z_{3}^{-1}{}^{{}^{2}}+Z_{3}^{1}{}^{{}^{2}}}}^{}$$

### Statistical Analysis

Data were analyzed using SPSS software version 23 (SPSS, Inc, Chicago, IL, USA). D’Agostino & Pearson Omnibus Normality Test was used to test whether the data were normally distributed. To compare logMAR, SE and astigmatism among the groups, one-way ANOVA with Tukey’s multiple comparisons post-test was performed. To compare the differences in HOAs and corneal opacity grading, the Kruskal Wallis test with Dunn’s multiple comparisons test was used. Spearman’s correlation analysis was used to evaluate the correlation between logMAR and HOAs of the total cornea within 4mm diameter. We carefully checked the medical records and slit-lamp findings in all patients and confirmed that patients with vision-affecting cataracts underwent cataract surgery. Patients with cataract (2 eyes in LCDIIIA), retinal disease (epiretinal membrane in 1 eye and diabetic macular edema in 1 eye in GCD2) and optic nerve atrophy (1 eye in GCD2) were excluded from the correlation analysis between logMAR and HOAs. Data were expressed as mean ± standard deviation (SD). A P-value of less than 0.05 was considered statistically significant.

## Results

### Patients Demographics

An overview of the study group characteristics is shown in Table 1. The mean age of the LCDI and MCD group were significantly younger than of the GCD2 group (P = 0.014). The mean age of the LCDIIIA group was significantly older than of the GCD2 group (P = 0.010). The mean values of logMAR in the LCDI and LCDIIIA group were significantly worse than that of the GCD2 group (P<0.001).

**Table 1:** Patients’ demographics.

	Normal	GCD2	LCDI	LCDIIIA	MCD	P*
**No of eyes**	18	121	20	32	13	
**Age (years)**	51.1 ± 18.5	60.0 ± 12.7	40.1 ± 20.2^†^	70.7 ± 10.7^††^	49.9 ± 5.8^†^	< 0.001
**Male / Female**	5/13	30 / 34	8 / 4	16 / 3	5 / 2	
**LogMAR**	-0.03 ± 0.05	0.20 ± 0.22	0.56 ± 0.49^‡^	0.71 ± 0.62^‡^	0.48 ± 0.62	< 0.001
**SE (D)**	-1.4 ± 2.9	-1.4 ± 3.0	-0.8 ± 1.9	0.0 ± 1.5	-0.7 ± 3.9	0.349
**Astigmatism (D)**	1.0 ± 0.7	1.1 ± 1.2	0.8 ± 0.9	1.1 ± 1.2	1.4 ± 1.1	0.582

GCD: granular corneal dystrophy, LCDI: lattice corneal dystrophy type 1, LCDIII: lattice corneal dystrophy type 3, MCD: macular corneal dystrophy, logMAR: the logarithm of the minimal angle of resolution, SE: spherical equivalent, D: diopter

*Kruskal-Wallis test

^†^P = 0.014, Dunn’s multiple comparison test, compared with the granular dystrophy

^††^P < 0.01, Dunn’s multiple comparison test, compared with the normal group and granular dystrophy

^‡^P < 0.001 Dunn’s multiple comparison test, compared with the granular dystrophy

### HOAs in Normal Subjects and Patients with Corneal Dystrophies

**Fig 1:**
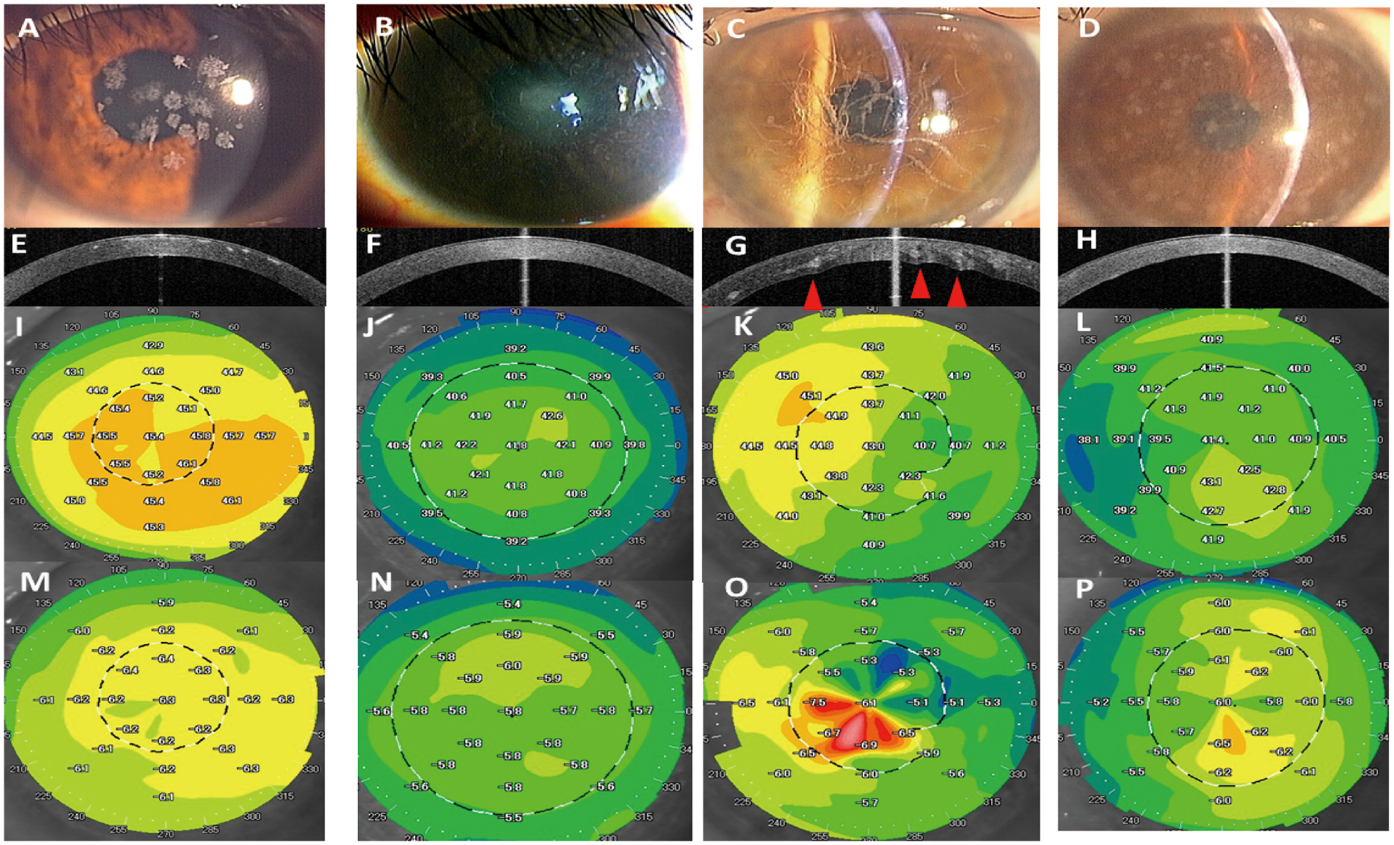
Representative cases of granular corneal dystrophy type2 (GCD2), lattice corneal dystrophy type 1 (LCDI), lattice corneal dystrophy type 3A (LCDIIIA) and macular corneal dystrophy (MCD) and the anterior segment optical coherence tomography (AS-OCT) images and topography maps of anterior and posterior corneal surfaces. The representative slit-lamp photographs (A-D), AS-OCT images (E-H), topography maps of anterior (I-L) and posterior (M-P) surfaces of GCD2 (A, E, I and M), LCDI (B, F, J and N), LCDIIIA (C, G, K and O), and MCD (D, H, L and P). In the slit-lamp photographs, the corneal opacities were scored as grade1 in GCD2 (A), grade 1 in LCDI (B), grade 2 in LCDIIIA (C) and grade 3 in MCD (D). In AS-OCT images and topography maps of GCD2, highly-reflective stromal opacities with clear boundary were embedded in the subepithelial and anterior stroma with minimal effects on anterior surface irregularities and no effects on posterior surface irregularities. AS-OCT images and topography maps in LCDI and MCD show diffuse clouding of full-thickness depth, with minimal irregularities in LCDI and moderate irregularities in MCD. In LCDIIIA, the irregularities of the posterior surface co-localize with lattice opacities in the deep stroma (red arrow heads).

The AS-OCT images show the differences in the smoothness of the posterior surface in each type of corneal dystrophy (Fig 1). The topography map of GCD2 show the minimal irregular astigmatism. However, the topography maps in LCDI, LCDIIIA and MCD show an asymmetric color map pattern for anterior and posterior surfaces. In LCDIIIA, the posterior surface was highly irregular (red arrowhead), and the topography maps of posterior corneal surface showed increased irregular astigmatism. The mean combined Zernike terms are shown in Table 2. Compared with the control subjects, subjects with GCD2, LCDI, LCDIIIA, and MCD showed significantly higher mean values for HOA and Coma terms of total, anterior, and posterior cornea in the central 4.0-mm and 6.0-mm zone (all Ps < 0.05). Compared with the control subjects, LCDI and LCDIIIA groups showed significantly higher values for all terms (all Ps < 0.05) except for the SA term of posterior cornea surface in the central 6.0-mm zone.

**Table 2:** Higher-order Aberration in Corneal Dystrophy.

	Control	GCD2	LCDI	LCDIIIA	MCD	P*	P^†^	P^†^	P^†^	P^†^
							^GCD2^	^LCDI^	^LCDIIIA^	^MCD^
**HOA (4mm)**										
**Total**	0.09 ± 0.02	0.17 ± 0.35	0.33 ± 0.27	0.61 ± 1.56	0.23 ± 0.18	<0.001	0.005	<0.001	<0.001	<0.001
**Anterior**	0.10 ± 0.02	0.17 ± 0.39	0.35 ± 0.29	0.51 ± 1.59	0.22 ± 0.18	<0.001	0.029	<0.001	<0.001	0.001
**Posterior**	0.02 ± 0.00	0.04 ± 0.05	0.05 ± 005	0.15 ± 0.16	0.04 ± 0.02	<0.001	<0.001	<0.001	<0.001	<0.001
**HOA (6mm)**										
**Total**	0.19 ± 0.06	0.32 ± 0.48	0.60 ± 0.46	0.83 ± 2.29	0.44 ± 0.24	<0.001	<0.001	<0.001	<0.001	<0.001
**Anterior**	0.21 ± 0.06	0.34 ± 0.50	0.63 ± 0.50	1.01 ± 3.22	0.44 ± 0.25	<0.001	0.014	<0.001	<0.001	<0.001
**Posterior**	0.06 ± 0.01	0.08 ± 0.05	0.09 ± 0.06	0.24 ± 0.19	0.09 ± 0.03	< 0.001	0.042	0.004	< 0.001	0.002
**SA (4mm)**										
**Total**	0.07 ± 0.02	0.12 ± 0.34	0.21 ± 0.16	0.51 ± 1.53	0.13 ± 0.10	<0.001	0.462	<0.001	<0.001	0.02
**Anterior**	0.08 ± 0.02	0.12 ± 0.38	0.21 ± 0.17	0.42 ± 1.52	0.13 ± 0.10	<0.001	0.999	0	0.003	0.096
**Posterior**	0.02 ± 0.00	0.03 ± 0.04	0.03 ± 0.02	0.15 ± 0.16	0.02 ± 0.01	<0.001	0.002	0.001	<0.001	0.214
**SA (6mm)**										
**Total**	0.13 ± 0.03	0.20 ± 0.38	0.30 ± 0.18	0.68 ± 2.24	0.22 ± 0.13	<0.001	0.239	<0.001	<0.001	<0.001
**Anterior**	0.14 ± 0.03	0.21 ± 0.41	0.31 ± 0.18	0.81 ± 3.15	0.22 ± 0.14	<0.001	0.397	<0.001	<0.001	0.186
**Posterior**	0.05 ± 0.01	0.06 ± 0.04	0.07 ± 0.04	0.16 ± 0.14	0.06 ± 0.02	< 0.001	0.173	0.412	< 0.001	0.331
**Coma (4mm)**										
**Total**	0.06 ± 0.02	0.11 ± 0.11	0.25 ± 0.24	0.27 ± 0.34	0.18 ± 0.15	<0.001	0.001	<0.001	<0.001	<0.001
**Anterior**	0.06 ± 0.02	0.11 ± 0.11	0.26 ± 0.25	0.25 ± 0.47	0.17 ± 0.16	<0.001	0.005	<0.001	<0.001	<0.001
**Posterior**	0.01 ± 0.00	0.03 ± 0.03	0.04 ± 0.04	0.16 ± 0.17	0.04 ± 0.02	<0.001	<0.001	<0.001	<0.001	<0.001
**Coma (6mm)**										
**Total**	0.14 ± 0.07	0.23 ± 0.31	0.50 ± 0.45	0.39 ± 0.52	0.38 ± 0.21	<0.001	0.02	<0.001	<0.001	<0.001
**Anterior**	0.15 ± 0.07	0.24 ± 0.30	0.52 ± 0.49	0.48 ± 0.78	0.38 ± 0.21	<0.001	0.03	0.003	<0.001	<0.001
**Posterior**	0.03 ± 0.02	0.04 ± 0.03	0.04 ± 0.04	0.17 ± 0.14	0.06 ± 0.02	< 0.001	0.043	0.013	< 0.001	0.001

Mean ± SD μm

HOA: higher-order aberration, SA: Spherical aberration, GCD2: granular corneal dystrophy 2, LCDI: lattice corneal dystrophy type I, LCDIIIA: lattice corneal dystrophy type IIIA, MCD: macular corneal dystrophy

*Kruskal Wallis test

^†^Dunn’s multiple comparison test, compared with healthy control

### HOAs Based on Corneal Opacity Grading

**Fig 2:**
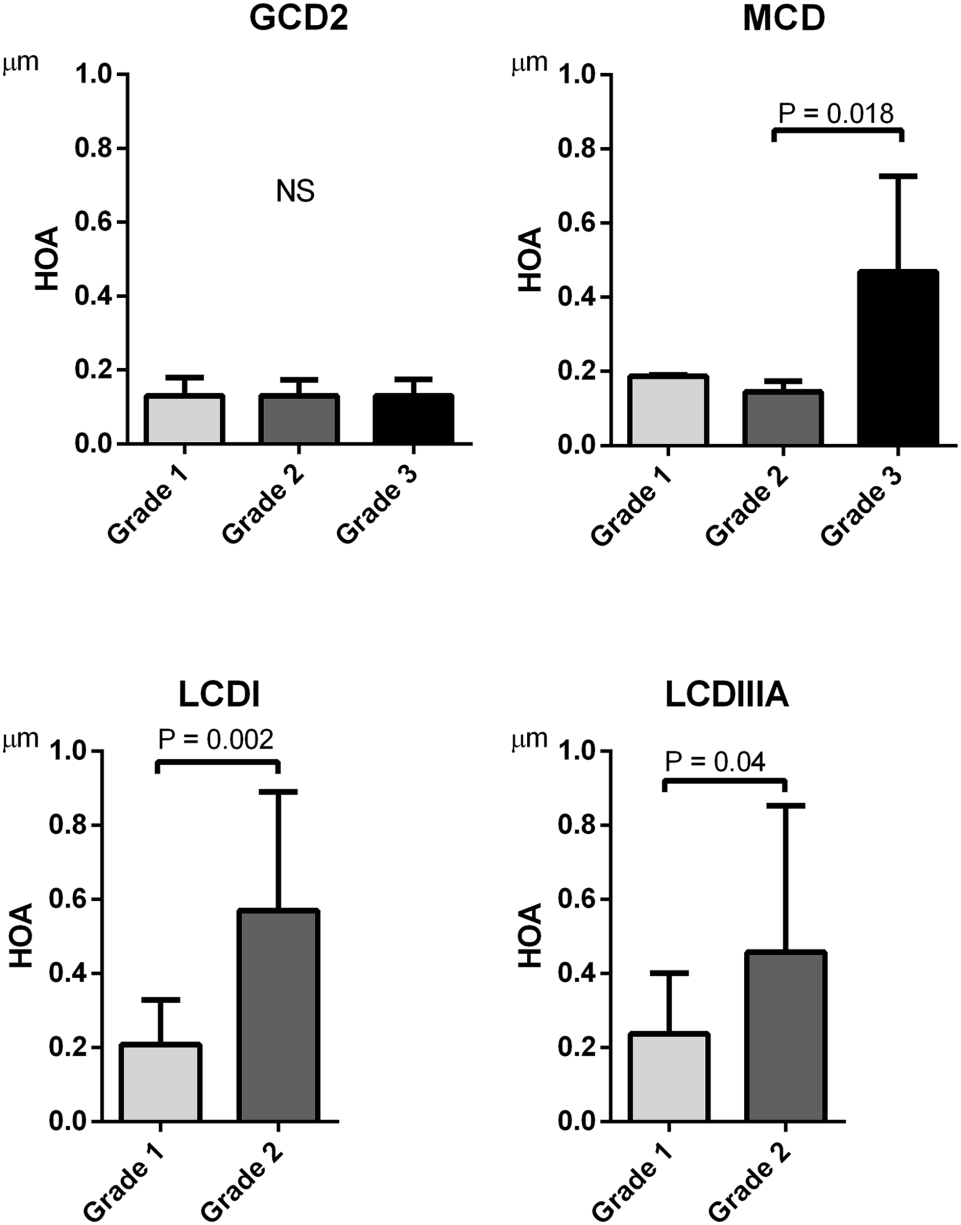
The higher-order aberrations (HOAs) based on corneal opacity grading in corneal dystrophies. There were no significant differences in HOAs among corneal opacity grade 1, 2 and 3 in GCD2 (all Ps > 0.05). In the MCD group, the HOAs in eyes with corneal opacity grade 3 were significantly larger than those of grade 2 (P = 0.018). In the LCDI and LCDIIIA group, the HOAs in eyes with corneal opacity grade 2 were significantly larger than those of grade 1 (P = 0.022 and P = 0.017, respectively).

The amounts of stratified HOAs based on the corneal opacity grading in each case of corneal dystrophy were analyzed (Fig 2). The values of HOAs in Fig 2 represent the HOAs of the total cornea within 4mm diameter. In GCD2, the HOAs did not increase with an increase in the corneal opacity grading. However, for the MCD group, the HOAs of eyes with grade 3 opacity significantly were significantly larger than those with grade 2 opacity, however there was no significant difference in HOAs between patients with grade 1 and grade 2. In the LCDI and LCDIIIA groups, the HOAs of eyes with grade 2 opacity were significantly larger than those with grade 1 opacity. However, there was no significant difference in HOAs between grade 2 and grade 3 opacities, which was possibly due to the small numbers of eyes with grade (3 eyes with grade 3 in LCDI, and 1 eyes with grade 3 in LCDIIIA)

### Correlation with Visual Acuity

In GCD2, the logMAR was positively correlated with opacity grade, age and astigmatism (Table 3 and supplementary table, r = 0.183, P = 0.047; r = 0.308, P = 0001; and r = 0.265, P = 0.004, respectively), whereas there was no significant correlation between HOA and logMAR in GCD2 (r = 0.113, P = 0.227). In contrast, in LCDI, the logMAR was significantly correlated only with HOA in the central 4.0 mm zone of the total cornea (r = 0.587, P = 0.007). In LCDIIIA and MCD, the logMAR was significantly correlated only with HOA in the central 4.0 mm zone of the total cornea (r = 0.614, P < 0.001, and r = 0.620, P = 0.028, respectively) and with corneal opacity grade (r = 0.487, P = 0.005, and r = 0.613, P = 0.026, respectively).

**Table 3:** Correlation analysis between logMAR and clinical parameters.

	GCD2	LCDI	LCDIIIA	MCD
**HOA (total 4mm)**	0.113	**0.587**	**0.614**	**0.62**
	-0.227	**-0.007**	**(<0.001)**	**-0.024**
**Opacity grade**	**0.183**	0.309	**0.487**	**0.613**
	**-0.047**	-0.185	**-0.005**	**-0.026**
**Age**	**0.308**	0.406	0.139	0.426
	**-0.001**	-0.075	-0.449	-0.146
**Spherical**	-0.131	0.234	-0.222	-0.311
	-0.157	-0.321	-0.222	-0.301
**Astigmatism**	**0.265**	0.36	0.35	0.086
	**-0.004**	-0.119	-0.05	-0.78

Spearman correlation analysis Correlation coefficient (P value)

GCD2: granular corneal dystrophy 2, LCDI: lattice corneal dystrophy type I, LCDIIIA: lattice corneal dystrophy type IIIA, MCD: macular corneal dystrophy

## Discussion

We reported increased HOAs in groups with LCDIIIA, LCDI, MCD, and GCD2 than in the normal group. In groups with MCD, LCDI, and LCDIIIA, HOAs increased as corneal opacity increased. We also showed that a larger HOA was associated with decreased visual acuity in groups with LCDI, LCDIIIA, and MCD, although there was no significant correlation between HOA and visual acuity in the GCD group.

Stromal corneal dystrophies are autosomal hereditary disorders characterized by bilateral progressive stromal opacity. Based on the AS-OCT images and topography maps in the current study, Fig 2 shows that there were clear differences in surface irregularities and stromal opacities. In GCD2, the highly reflective stromal opacities with clear boundary were embedded in the subepithelial and anterior stroma with minimal effect on anterior and posterior surface irregularities. The stromal opacities in LCDI and MCD were characterized by the diffuse stromal clouding with moderate effects on the irregularities of both the anterior and posterior surfaces. In LCDIIIA, the lattice-shaped stromal opacities with various reflectivities, sizes and depths had a direct effect on the irregularities of both the anterior and posterior surfaces. We quantified the corneal HOAs of corneal dystrophies and found that the amount of HOAs in both anterior and posterior surfaces were larger in corneal dystrophies than control subjects. Furthermore, the patterns and amounts of HOAs varied among the types of corneal dystrophies. The increase in SA of both anterior and posterior surfaces were minimal in GCD2 and MCD, moderate in LCDI, and large in LCDIIIA. Compared with normal control subjects, the amounts of coma aberrations were moderate in GCD2 and MCD and large in LCDI and LCDIIIA groups.

The association of HOAs and corneal opacity varied among the corneal dystrophies. The amount HOAs did not change with corneal opacity in GCD, however, increased from the early stage of the disease in LCDI and LCDIIIA (corneal opacity grade 1 to grade 2). In contrast, the HOAs increased at the late stage of the disease in MCD (corneal opacity grade 2 to grade 3). These results indicate that stromal opacity in GCD2 does not influence the corneal structures and curvature. In contrast, advanced stromal opacity in LCD and MCD altered the microstructure of the corneal stroma, changing the curvatures of the corneal anterior and posterior surfaces and inducing larger amount of HOAs.

With regards to surface irregularities and corneal HOAs in corneal dystrophies, Seitz et al. reported that one of the goals of PTK is “to regularized the surface and treat irregular astigmatism”.[20] Excimer laser PTK is known to be a safe and minimally invasive treatment of corneal dystrophies, effective in delaying or avoiding corneal transplantation.[21–23] Nassaralla et al. reported a series of four eyes with GCD and LCD with no recurrence subsequent to PTK with follow-ups of 36–58 months.[24] Das et al. reported that the BCVA improved in 79% of 50 patients with GCD and 62% of 12 patients with LCD after PTK.[21] The smaller percentage in improvement for LCD patients compared to GCD patients may be due to the difference in the depth of the opacities and HOAs of the posterior surfaces, as reported by Seitz et al. after histologically comparing these two different dystrophies.[25]

The non-invasive objective assessment of corneal HOAs using AS-OCT has great potential to increase our understanding pertaining to the interaction among visual impairment, visual symptomatology and timing of corneal surgeries. To optimize the efficacy of PTK, the custom ablation of the anterior stroma, together with the removal of anterior stromal opacities, represents a promising approach for decreasing HOAs of the anterior surface. However, the amount of HOAs of the posterior surface are as large as 0.24 μm for 6mm diameter in LCD IIIA patients, whereas the amount of HOAs of the posterior surface are relatively small (from 0.08 to 0.09 μm for 6mm diameter) in GCD2, LCDI and MCD. If the amount of HOAs of the posterior surface is as large as that found in LCDIIIA patients with impaired visual function, the corneal transplantation can be one of the first options for improving visual acuity.

The HOAs of total cornea were significantly correlated with visual acuity in groups with LCD and MCD, whereas there was no correlation between HOAs and visual acuity in GCD group. This could be partly due to the small amount of HOAs in GCD group, and the possibility that the degradation of visual function depends more on stromal opacities in GCD. In LCD and MCD, HOAs together with stromal opacities are thought to have serious effects on visual acuity. With regards to clinical visual optics, Applegate et al. reported that the interaction of HOAs can improve or degrade retinal image quality.[26, 27] However, it is assumed that visual function of patients with corneal dystrophies correlates with HOAs, as reported in previous studies.[6, 8–11, 14, 15]

Corneal HOAs can be measured using several techniques, including the Hartman-Shack aberrometer, the Scheimpflug camera system and AS-OCT. However, whole eye evaluations can be influenced by the corneal opacity, the HOAs of lens and accommodation.[28] Because our objective was to characterize the HOAs of total cornea, plus the anterior and posterior surfaces in eyes with stromal opacities, we thought the Scheimpflug camera system or AS-OCT are applicable for this study. The Scheimpflug camera system obtains corneal data using a short wavelength of 475 nm, whereas the AS-OCT uses a longer wavelength of 1310nm. The longer wavelength has advantage of deeper penetration with less reflection in eyes with corneal opacities. Fukuda et al found good repeatability coefficients by using the AS-OCT system. Therefore, in this study, we used the AS-OCT system. [29]

This study has several limitations. First, these corneal dystrophies induce forward and backward scattering as a result of the accumulation of corneal opacities. To evaluate the effect of light scattering on visual function, we will need to apply new technologies, such as the optical quality analysis system (OQAS, Visiometrics, Terrassa, Spain) or the cataract quantifier (C-Quant, Oculus, Wetzlar, Germany) in future comprehensive studies. Second, the average ages of the patients with corneal dystrophies were different, which may have introduced bias. Age has been reported to be strongly correlated with visual function, refraction, astigmatism and HOAs.[8, 30, 31] As previously reported, the onset of corneal opacity varies among corneal dystrophies. Musch et al. reported that the average age of MCD was significantly younger than that of LCD.[32] Thus, age can be a confounding factor because stromal opacities increase with age in corneal dystrophies. In the current study, age showed no significant correlation with HOAs and logMAR for groups with LCD and MCD, whereas age was positively correlated with logMAR only in the GCD2 group (S1 Tables). We postulate that the increase of HOAs was smallest in GCD2, compared with LCD and MCD, which minimizes the influence of HOAs on visual acuity in GCD2. In groups with LCDI, LCDIIIA and MCD, HOA was significantly correlated with visual acuity, and not with age, suggesting that the assessment of HOA can provide a good index for explaining decreased visual acuity and the degree of stromal opacities in LCD and MCD. The other limitation of this study is that we assumed that normal cornea and cornea with stromal opacity had the same refractive index, which may have influenced our estimate of HOAs. The corneal deposits of corneal dystrophies can affect refractive index, although the effect is still poorly understood.

In conclusion, we found that HOAs of total cornea, and anterior and posterior surfaces are larger in subjects with corneal dystrophies than normal control subjects, suggesting that the stromal opacities of the LCD, GCD2 and MCD induce not only scatter but also morphometric changes in anterior and posterior surfaces that can cause HOAs. Furthermore, there are differences in the amount of corneal HOAs among corneal dystrophies. Increases of HOAs are minimal in GCD2, but greater for LCD and MCD. The HOAs are significantly correlated with visual acuity for patients with LCD and MCD, but not for those with GCD2.

## Supporting Information

S1 Tables Correlations among visual acuity, age, spherical equivalent, corneal opacity grade and HOA in GCD2, LCDI, LCDIIIA and MCD. S1 tables show the correlation coefficients and P values among visual acuity, age, spherical equivalent, corneal opacity grade and HOAs in GCD2, LCDI, LCDIIIA and MCD.
(DOCX)

S1 Dataset (XLSX)
