# Supplementary material for: Corneal Higher Order Aberrations in Granular, Lattice and Macular Corneal Dystrophies
Source: PLoS One. 2016 Aug 18;11(8):e0161075. doi: 10.1371/journal.pone.0161075 (PMC4990250; doi:10.1371/journal.pone.0161075)
Supplement: S1 Tables — show the correlation coefficients and P values among visual acuity, age, spherical equivalent, corneal opacity grade and HOAs in GCD2, LCDI, LCDIIIA and MCD. (DOCX) [file pone.0161075.s001.docx]

**S1 tables. Correlation among visual acuity, age, spherical equivalent, corneal opacity grade and HOA**

**GCD2**

|  | | **LogMAR** | **Age** | **SE** | **Astigmatism** | **Corneal opacity grade** | **HOAs** |
| --- | --- | --- | --- | --- | --- | --- | --- |
| **LogMAR** | r | 1.000 | **.308^**^** | -.131 | **.265^**^** | **.183^*^** | .114 |
|  | P value | . | **.001** | .157 | **.004** | **.047** | .216 |
| **Age** | r | **.308^**^** | 1.000 | **.375^**^** | **.348^**^** | .123 | .002 |
|  | P value | **.001** | . | **.000** | **.000** | .184 | .981 |
| **SE** | r | -.131 | **.375^**^** | 1.000 | -.059 | -.032 | **-.195^*^** |
|  | P value | .157 | **.000** | . | .526 | .733 | **.033** |
| **Astigmatism** | r | **.265^**^** | **.348^**^** | -.059 | 1.000 | .020 | .032 |
|  | P value | **.004** | **.000** | .526 | . | .825 | .730 |
| **Corneal opacity grade** | r | **.183^*^** | .123 | -.032 | .020 | 1.000 | -.062 |
|  | P value | **.047** | .184 | .733 | .825 | . | .505 |
| **HOAs** | r | .114 | .002 | **-.195^*^** | .032 | -.062 | 1.000 |
|  | P value | .216 | .981 | **.033** | .730 | .505 | . |

Spearman’s correlation analysis

GCD: granular corneal dystrophy, SE: spherical equivalent, HOAs: higher order aberrations

**LCDI**

|  | | **LogMAR** | **Age** | **SE** | **Astigmatism** | **Corneal opacity grade** | **HOAs** |
| --- | --- | --- | --- | --- | --- | --- | --- |
| **LogMAR** | r | 1.000 | .406 | .234 | .360 | .309 | **.587^**^** |
|  | P value | . | .075 | .321 | .119 | .185 | **.007** |
| **Age** | r | .406 | 1.000 | .438 | .414 | .406 | .247 |
|  | P value | .075 | . | .053 | .069 | .076 | .293 |
| **SE** | r | .234 | .438 | 1.000 | .408 | .103 | -.092 |
|  | P value | .321 | .053 | . | .075 | .666 | .700 |
| **Astigmatism** | r | .360 | .414 | .408 | 1.000 | .502^*^ | .082 |
|  | P value | .119 | .069 | .075 | . | .024 | .731 |
| **Corneal opacity grade** | r | .309 | .406 | .103 | .502^*^ | 1.000 | **.607^**^** |
|  | P value | .185 | .076 | .666 | .024 | . | **.005** |
| **HOAs** | r | **.587^**^** | .247 | -.092 | .082 | **.607^**^** | 1.000 |
|  | P value | **.007** | .293 | .700 | .731 | **.005** | . |

Spearman’s correlation analysis

LCD: lattice corneal dystrophy, SE: spherical equivalent, HOAs: higher order aberrations

**LCDIIIA**

|  | | **LogMAR** | **Age** | **SE** | **Astigmatism** | **Corneal opacity grade** | **HOAs** |
| --- | --- | --- | --- | --- | --- | --- | --- |
| **LogMAR** | r | 1.000 | .139 | -.222 | .350 | **.487^**^** | **.614^**^** |
|  | P value | . | .449 | .222 | .050 | **.005** | **<.001** |
| **Age** | r | .139 | 1.000 | -.070 | .158 | -.029 | .020 |
|  | P value | .449 | . | .704 | .387 | .875 | .914 |
| **SE** | r | -.222 | -.070 | 1.000 | -.111 | .030 | -.063 |
|  | P value | .222 | .704 | . | .545 | .872 | .732 |
| **Astigmatism** | r | .350 | .158 | -.111 | 1.000 | .296 | .270 |
|  | P value | .050 | .387 | .545 | . | .100 | .135 |
| **Corneal opacity grade** | r | **.487^**^** | -.029 | .030 | .296 | 1.000 | **.522^**^** |
|  | P value | **.005** | .875 | .872 | .100 | . | **.002** |
| **HOAs** | r | **.614^**^** | .020 | -.063 | .270 | **.522^**^** | 1.000 |
|  | P value | **<.001** | .914 | .732 | .135 | **.002** | . |

Spearman’s correlation analysis

LCD: lattice corneal dystrophy, SE: spherical equivalent, HOAs: higher order aberrations

**MCD**

|  | | **LogMAR** | **Age** | **SE** | **Astigmatism** | **Corneal opacity grade** | **HOAs** |
| --- | --- | --- | --- | --- | --- | --- | --- |
| **LogMAR** | r | 1.000 | .426 | -.311 | .086 | **-.613^*^** | **-.620^*^** |
|  | P value | . | .146 | .301 | .780 | **.026** | **.024** |
| **Age** | r | .426 | 1.000 | -.064 | **.624^*^** | .080 | .055 |
|  | P value | .146 | . | .836 | **.023** | .794 | .857 |
| **SE** | r | -.311 | -.064 | 1.000 | -.036 | .043 | .515 |
|  | P value | .301 | .836 | . | .906 | .889 | .072 |
| **Astigmatism** | r | .086 | **.624^*^** | -.036 | 1.000 | .394 | .070 |
|  | P value | .780 | **.023** | .906 | . | .183 | .821 |
| **Corneal opacity grade** | r | **-.613^*^** | .080 | .043 | .394 | 1.000 | .340 |
|  | P value | **.026** | .794 | .889 | .183 | . | .256 |
| **HOAs** | r | **-.620^*^** | .055 | .515 | .070 | .340 | 1.000 |
|  | P value | **.024** | .857 | .072 | .821 | .256 | . |

Spearman’s correlation analysis

MCD: macular corneal dystrophy, SE: spherical equivalent, HOAs: higher order aberrations
